# Effects of Body Mass Index (BMI), demographic and socioeconomic factors on organized physical activity (OPA) participation in children aged 6-15 years: a cross-sectional study comparing primary and secondary school children in Greece

**DOI:** 10.1186/s12887-020-02276-6

**Published:** 2020-10-22

**Authors:** Vilelmine Carayanni, Elpis Vlachopadopoulou, Dimitra Koutsouki, Gregory C. Bogdanis, Theodora Psaltopoulou, YannisManios, Feneli Karachaliou, Angelos Hatzakis, Stefanos Michalacos

**Affiliations:** 1 Laboratory of Statistical Modelling and Educational Technology in Public and Environmental Health– sepeh.lab, Department of Public and Community Health, University of West Αttica, 28 Saint Spyridonos str, 12243 Egaleo, Greece; 2 Department of Endocrinology, Children’s Hospital P. & A. Kyriakou, Thibon & Levadeias str, Ampelokipoi T.K, 11527 Athens, Greece; 3 School of Physical Education & Sports Science, National and Kapodistrian University of Athens, 41, Ethnikis Antistaseos str., 17237 Daphne, Athens, Greece; 4 Department of Hygiene, Epidemiology and Medical Statistics, School of Medicine, National and Kapodistrian University of Athens, 75 Mikras Asias str, 11527 Goudi, Greece; 5 Department of Nutrition & Dietetics, School of Health Science & Education, Harokopio University, 70, El Venizelou Ave 176 71 Kallithea, Athens, Greece

**Keywords:** Organized sports participation, Body mass index (BMI) category, Obesity, Primary and secondary schools, Socioeconomic status index (SES), Greece

## Abstract

**Background:**

The aim of the present study was to examine the influence of body mass index category, as well as of demographic and socioeconomic factors on the participation in organized physical activity (OPA) of schoolchildren attending primary and secondary school in Greece. Furthermore, to compare the difference between the two levels.

**Methods:**

This is a cross-sectional study conducted on a representative elementary and secondary school cohort, derived using stratification and probability proportional to size (PPS) methodology. The final sample included 18,264 subjects, aged 6 to 15 years.

Parents of all students and students of secondary schools fulfilled validated questionnaires evaluating socioeconomic status, and participation to OPA. International Obesity Task Force (IOTF) cut offs were used to classify the children. Univariate and multivariate logistic models examined factors associated with OPA. All analyses were stratified by school level.

**Results:**

Sport participation was not reported in 37.7 and 54.4% of primary and secondary schoolchildren respectively. Having BMI within normal range, being male, having parents participating in an organized activity and a high socio-economic status seem to encourage participation in OPA in both school levels.

**Conclusions:**

Children with normal BMI are more likely to participate in OPA. Parents as role model as well as higher socioeconomic status of the family emerge as important influencers. Participation in OPA declines as students enter secondary school. Interventions focusing on increasing physical education and activity into school daily program should be tailored to the specific needs of different weight categories and can possibly eliminate the impact of SES inequalities.

## Background

Low levels of physical activity and excessive sedentary time in childhood are associated with short and long-term psychological and physiological consequences [1, 2]. Physical inactivity, is an important factor that may contribute to low aerobic fitness, as well as to low energy expenditure, which may result in excessive weight gain [3]. In contrast, increased physical activity levels have been recognized as an important factor for the prevention and treatment of obesity [4]. Participation of young children and adolescents in organized sport training increases their physical activity level and contributes towards achieving the desired daily and weekly physical activity targets [5–8]. Furthermore, participation in competitive sports at club level, has been shown to increase the chances of reaching healthy cardiorespiratory fitness levels [9]. Organized physical activities (OPA) comprise exercise programs in specially designed areas, tend to require a coach or an instructor, are structured, and require payment [10]. Participation in OPA has been shown to be positively associated with proficiency of fundamental movements and physical fitness [11], because of the continuous guidance and errors correction by a coach/instructor and the latter’s systematic structured content. Also, training exercises are individualized according to a person’s abilities.

There is evidence that girls are less active than boys [12, 13]. Additionally, socio-demographic factors such as age and geographic area (rural area),seem to constitute risk factors for non participation in OPA [14, 15].There is a number of studies showing that socioeconomic status, BMI category and family settings play an important role in regards to participation in OPA [6, 8, 16, 17]. Another important factor influencing OPA may be the transition from primary to secondary school. This transition involves a significant life change and might be accompanied with a significant decrease in sport participation [18].

Thus, the aim of the present study was to examine the influence of BMI category, as well as demographic and socioeconomic factors on the participation in OPA in a large and representative sample of schoolchildren attending primary and secondary schools in Greece and compare the two levels.

## Methods

### Sampling and participants

The “Hellenic Action Plan for the Assessment, Prevention and Treatment for Childhood Obesity” was a school-based survey, financed by the National Strategic Reference Framework, conducted at a national level in Greece and provided the data that were used in the present analysis. Approval to conduct the study was granted by the Greek Ministry of National Education and the Greek Ministry of Health. Data were collected between 1/2015 and 6/2015. The study population comprised schoolchildren attending all grades of primary and secondary schools located in several municipalities in Greece (including rural areas and islands). **Probability proportional** to **size (PPS)** sampling was applied. The sampling of schools was stratified by the type of school, (primary or secondary), proportionally to the total number of pupils attending these schools. Following this procedure, an appropriate number of schools were randomly selected from each one of these municipalities, specifically, 278 schools, i.e. 205 primary and 73 secondary schools, were included. Prior to acceptance of children’s participation to the study, an extended letter explaining the objectives of the study were provided to all parents or guardians whose children were attending these schools. Parents who approved participation of their children to the study have signed an informed consent. The response rate was 71% as 18,307 parents out of the 25,942 who signed parental consent forms have fully completed the questionnaire. After examination of univariate statistics to detect any anomalies [19] in the distribution of variables, (especially outliers or missing values 10 values on the total), aberrant values and duplicates (33 cases on the total), the total study sample in this analysis included 18,264 children aged between 6 to 15 years, who had complete physical activity data. The larger part of this sample (72.0% or 13,119 children) were primary school pupils, (ages 6–12), while 5145 (28.0%) were secondary school (ages 12–15) pupils. Pupils with severe chronic illnesses i.e. malignancies, diabetes mellitus, rheumatoid arthritis or systemic lupus erythematosusor receiving chronic therapies for more than 6 months per year for any diagnosis, were excluded from the analysis (70 children in primary schools and 43 children in secondary schools).

### Μeasurements

Anthropometric measurements were conducted by sixteen health professionals, specially hired and trained for the purposes of the present study. Children were measured by two trained members of the research team. The protocol and equipment used were common in all schools. Each child was measured three times, and the average of the three measurements was computed. Body weight was measured to the nearest 100 g using a Tanita digital scale (Tanita BWB 800ΜΑ). Pupils were weighed without shoes with minimal possible clothing. Height was measured to the nearest 0.1 cm using a commercial stadiometer (Charder HM 200P Portstad). The Charder stadiometer was standardized against a Harpenden portable stadiometer. During the measurement of height, each pupil was standing barefoot, keeping shoulders in a relaxed position, arms hanging freely and head aligned to the Frankfort horizontal plane. Body weight and height were used to calculate BMI, using the Quetelet’s equation, i.e. body weight (kg) / height^2^ (m^2^). BMI was calculated and subsequently participants were categorized according to the IOTF criteria [20] into the following four BMI categories: Underweight, Normal, Overweight and Obese. Both interrater and intra-rater reliability, measured with the intraclass correlation coefficients yielded values greater than 0.97.

### Measures

Three structured questionnaires were developed and administered to the parents and students. In elementary school the parents filled the questionnaires and in high schools two different questionnaires were answered by the students and the parents respectively. Validity and reliability of the three questionnaires was tested and found to satisfy the principles of reliability and validity [21]. In order to assess reliability and validity, prior to initiation of the study, data were collected from 450 parents of children aged 6–12 (n_1_), 450 parents of children aged 12–15 (n_2_), and 250 adolescents aged 12–15 (n_3_), from the region of Attica. Exploratory factor analysis (EFA) was performed and Confirmatory Factor Αnalysis (CFA) followed in another sample (n_1_ = 163, n_2_ = 163, n_3_ = 93) from 3 different regions to verify the factor structure. Cronbach’s alpha (α) and Intraclass Correlation Coefficients (ICC) were used to test reliability. EFA results support the factorability of the correlation matrix. (Kaiser-Meyer-Oklin: 0.941–0.944 and Bartlett’s test of Sphericity< 0.001 in all cases). CFA revealed that the final models (6-factor models for parents and 5-factor models for adolescents) provided the best fit for our samples (RMSEA: 0.04–0.05 and CFI:O.90–0.94**).** Cronbach’s alpha and ICC showed acceptable reliability (α: 0.79 = 0.90 and ICC:> 0.67–0.91). All questionnaires were granted clearance by the Hellenic Data Protection Authority. The factors addressed included socio-demographic characteristics, such as socio-economic status, age, gender, geographic area (urban, semi urban, rural),and OPA habits. Semi-urban population includes the population of municipalities and communities, whose most populous settlement has 2000–9999 inhabitants, except those belonging to urban planning.

SES index is a score that characterizes socioeconomic status. It is derived by the combination of 5 factors, precisely, mother’s and father’s years of education, number of cars, rent or owned house and surface area of residence per number of inhabitants, composing the SES index that has a range between of 0–13, with higher values indicating higher SES of the family.as previously published by Moschonis et al., 2013 [22].

With regard to the participation in OPA which is the outcome of the present analysis, a special section of the questionnaire addressed the participation in out-of-school organized sports activities defined as structured in organized athletic facilities under the auspices of the state, athletic federations and sports and athletic clubs officially registered in the General Secretariat for Sports, that belongs to the Greek Ministry of Education.

.

### Statistical analysis

The outcome of the study was the prevalence of children participation in OPA, in relation to BMI category, socioeconomic factors, and different socio-demographic variables. For the purpose of analysis, the variable “OPA” was dichotomized as “Yes—participated in organized team or individual sports at least twice per week” and “No—did not participate in organized teams or individual sports at least twice per week since September of the current school year”. Examples were basketball, football,volleyball, swimming, dance, aerobics martial arts tennis, track and field and other. Due to the sampling procedure described above, the present data yield robust estimates of the prevalence of the four BMI categories in this cohort. Therefore, the prevalence is reported together with its 95% confidence intervals (CI).

Descriptive statistics were performed. Also, the χ^2^ statistical test and the Mann-Witney test were used to test the homogeneity between primary and secondary schoolchildren in variables of interest. Two-step cluster analysis procedure was done to explore SES grouping of participants [22]. Number of clusters was limited to three (high, moderate, and low SES). Univariate and multivariate logistic models were used to examine the association of the above mentioned factors with OPA, by school level.

The variables found, by use of univariate analysis, to be associated with the outcome variable at the *p <* 0.10 level were included in the initial models to determine which factors were independent predictors of the outcome variable in the study subjects. Sandwich Variance Estimators were used in order to provide a consistent estimate of the variance-covariance matrix of parameter estimates [23]. A full model with main effects was fitted, and backward elimination was used to obtain a reduced model. The variables associated with the outcome variable at *<* 0.05 level were maintained in the final models. Information criterion tests such as Akaike Information Criterion (AIC) and Bayesian Information Criterion (BIC) are used to compare models and select the better fitted model [24, 25].

All analyses were stratified by school level comparing primary and secondary schools. The results were recorded as frequencies (N) and percentages, means and standard deviations (SD), unadjusted and adjusted, odds ratios (OR) with 95% confidence intervals (CI), and *p* values.

Statistical analyses were carried out using SPSS (IBM SPSS Statistics Version 23) and R language (Version 3.2.3).

## Results

### Sample characteristics and SES results

**Fig. 1:**
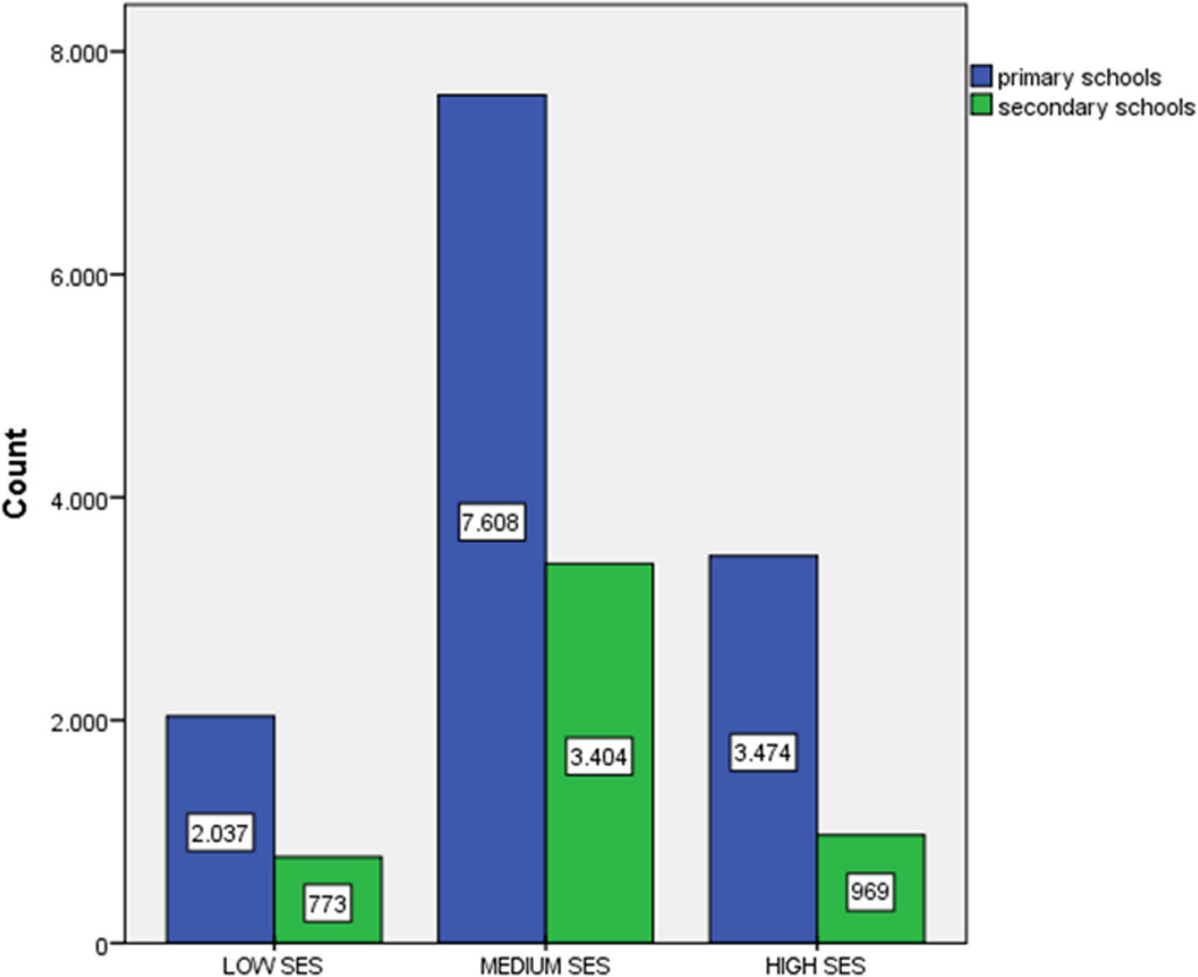
SES distribution by School type

Missing values do not surpass 10% in any variable measured. Descriptive and inferential statistics for the distribution of socioeconomic and demographic variables in our sample are given in Tables 1 and 2. As can be seen (Table 1), the mothers of primary schoolchildren presented in general a higher educational level than those of secondary schoolchildren (*p* = 0.04) and the primary schoolchildren families presented a higher household size (m^2^) per family member (*p* < 0.001). Nο significant differences were observed between primary and secondary schools in any other SES variables. Clusters distribution is presented in Fig. 1: The largest cluster that represents the medium SES (mean: 6.9, SD: 0.6) has 60.3% of the clustered cases (58.0% for primary schools and 66.2% for secondary schools). Also, the mean SES was significantly higher (*p* < 0.001) in primary schoolchildren families (mean: 6.9, SD: 1.6) compared to secondary schoolchildren families (mean: 6.6, SD: 1.5).

**Table 1:** Descriptive statistics and tests results for socioeconomic status (SES) in children aged 6-15 years (*N* = 18,264), by School level (ptimary and secondary schoolchildren)

Socio-economic status variables	Total N(%)	Primary schoolchildre N(%)	Secondary schoolchildren N (%)	*P*-value
Years of Paternal education				
< 9 years	1830(7.60)	1146(10.10)	684(15.70)	0.33**
9–12 years	8499(56.60)	6145(54.10)	2354(54.00)	
12–16 years	5002(33.30)	3761(33.20)	1241(28.40)	
> 16 years	382(2.50)	298(2.60)	84(1.90)	
Years of Maternal education				
< 9 years	1420(10.60)	889(7.10)	531(11.80)	< 0.04**
9–12 years	7971(53.90)	5579(47.90)	2392(53.30)	
12–16 years	6383(33.10)	4906(42.10)	1477(33.00)	
16 years	359(2.40)	275(2.40)	84(1.90)	
Socio-economic status variables	Total N(%)	Primary schoolchildren N(%)	Secondary schoolchildren N (%)	*P* value
Ηome square meters (m^2^)/ persons living at home				
< 50	16,878 (98.00)	11,944 (96.60)	4934 (100.00)	< 0.001*
50–75	395 (2.00)	395 (3.00)		
76–130	26 (< 0.10)	26 (0.20)		
Number of cars				
0	934(5.30)	617(4.90)	317(6.30)	0.19**
1	8705(49.10)	645(48.50)	2560(50.70)	
2	7364(41.60)	5427(42.80)	1937(38.40)	
≥ 3	717(4.00)	482(3.80)	235(4.70)	
Home property				
Rent	1821(10.30)	1029(8.10)	792 (15.40)	0.14*
Owned	15,879(89.70)	11,746(91.90)	4133(80.30)	

*The Chi-square test was used to calculate the *p*-value

**The Mann-Whitney test was used to calculate the *p*-value

**Table 2:** Descriptive statistics and statistical tests results for sociodemographic characteristics of children attending primary and secondary schools

Variables	Total N(%)	Primary schoolchildren N(%)	Secondary schoolchildren N (%)	*p* value
Gender				
Girl	9489 (50.40)	6621 (50.50)	2868 (51.00)	0.65*
Boy	9332 (49.60)	6483 (49.50)	2849 (49.00)	
Participation in OPA				
Yes	10,519(57.60)	8171(62.3)	2348(44.60)	< 0.001*
No	7747(42.40)	4948 (37.7)	2799(55.40)	
BMI				
Underweight	427 (2.50)	57(0.50)	370(7.40)	< 0.001**
Normal weight	10,660(62.90)	7607(63.40)	3053(61.00)	
Overweight	4296 (25.30)	3057(25.80)	1235(24.00)	
Obese	1573 (9.30)	1229(10.40)	339(6.60)	
Father’s participation in OPA				
Yes	8821(48.30)	6628(50.50)	2193(42.60)	< 0.001*
No	9443(51.90)	6491(49.50)	2952(57.40)	
Μother’s participation in OPA				
Yes	9957(56.30)	7038(53.60)	1646(32.00)	0.03*
No	7727(43.70)	6081 (46.40)	2919(56.70)	
Geographic Area				
Rural	5510 (30.20)	4267 (32.50)	1243 (24.10)	0.20**
Semi Urban	4797 (26.20)	3843(29.30)	954(18.50)	
Urban	7957 (43.60)	5009(38.20)	2948(57.40)	

*The Chi-square test was used to calculate the *p*-value

**The Mann-Whitney test was used to calculate the *p*-value

Τhe prevalence of participation in OPA (Table 2) was 57.6% (95% CI: 55.4–59.7%). More analytically, the prevalence of participation in OPA was 62.3% (95% CI: 61.3–63.7%) in primary schools and 44.6% (95% CI: 42.7–47.4%) in secondary schools, reflecting a dramatic decrease in OPA participation from primary to secondary school and these differences were highly significant (*p* < 0.001) (Table 2). Fifty five percent of the children (58.0%in primary and 48.0% in secondary school children) who reported participation in an OPA, practiced sports in Sports Clubs, 16.0% (13.0% in primary and 23.0% in secondary school children) in a Gym, and 14.0% (13.0% in primary and 15.0% in secondary school children) inprograms organized by the municipality. Also, significant differences (*p* < 0.001) were detected in BMI categories between primary and secondary schools, with primary schoolchildren being more likely to be overweight (25.8% vs 24.0%) and obese (10.4% vs 6.6%) and not underweight (0.5% vs 7.4%) compared to secondary schoolchildren.

Highly significant differences were also observed in parents’ participation in OPA between primary and secondary schools. Furthermore, there was a dramatic decrease in parents’ OPA from primary to secondary school and these differences were highly significant, especially in case of father’s participation in an OPA (p < 0.001).

### Univariate models

Influence of BMI category.

**Fig. 2:**
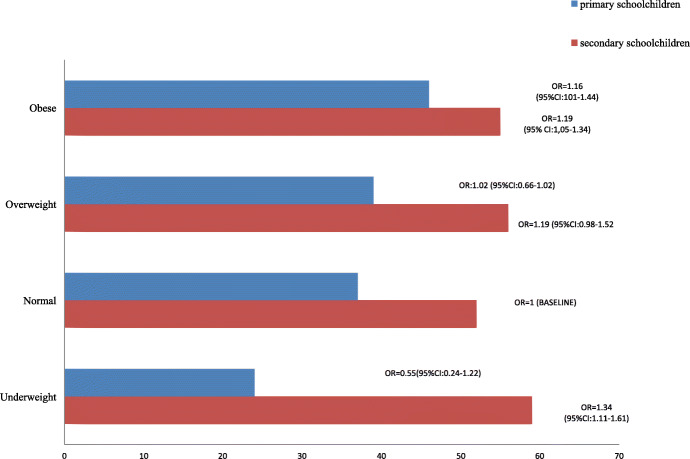
% Proportion of children and adolescents with no reported organized sports participation for each BMI category and Odds Ratios (baseline = normal weight)

Underweight children participated more frequently in an OPA (75.8% vs 52.0% in normal weight children) than normal weight children (Fig. 2), although this association isn’t statistically significant (*p* = 0.70). On the other hand, underweight adolescents were 1.34 times (95%CI: 1.11–1.61) more likely to not participate in an OPA than normal weight children.

Overweight primary schoolchildren participated less frequently in an OPA than normal weight children (39% vs 37% respectively).

. Results for overweight secondary schoolchildren pointed in the same direction, (56% of overweight adolescents vs 52% of normal weight adolescents), but none of these differences was statistically significant (*p* > 0.05).

Obese primary schoolchildren were 1.19 times (95% CI:1.05–1.34) more likely not to participate in an OPA (46% vs 37% respectively) than normal weight children. Also, obese secondary school children were 1.16 times (95%CI: 1.01–1.44) more likely not to participate in an OPA, (55% vs 52% respectively), than normal weight children (Fig. 2).

### Influence of sociodemographic factors

Table 3 indicates the significant results (*p*<0.10), at least for one school level, from univariate analysis of the above mentioned socio-demographic and demographic characteristics associated with organized activity participation, by school level.

**Table 3:** Significant results for at least one school level from univariate analysis and interactions effects analysis of covariates associated with prevalence of OPA in the primary and secondary schools children, Greece

	*P* value		OR (95% CI)	
Variables	Primary schoolchildren	Secondary schoolchildren	Primary schoolchildren	Secondary schoolchildren
Gender (males vs females)	< 0.001	< 0.001	1.25 (1.16–1.34)	1.16(1.04–1.30)
Father’s participation in OPA (yes vs no)	< 0.001	< 0.001	1.51 (1.41–1.62)	1.24 (1.10–1.39)
Mother’s participation in OPA (yes vs no)	< 0.001	0.01	1.32 (1.18–1.47)	1.176 (1.05–1.32)
Obesity (vs normal weight)	0.03	0.04	0.83 (0.74–0.95)	0.87 (0.77–0.98)
Overweight (vs normal weight)	0.17	0.06	0.84 (0.66–1.02)	0.80 (0.64–1.01)
Underweight (vs normal weight)	0.70	0.03	1.82 (0.81–4.16)	0.75 (0.62–0.90)
Geographic area (Urban vs rural)	0.32	< 0.001	0.92 (0.52–1.04)	1.60 (1.486–1.713)
Low SES (vs other categories)	< 0.001	< 0.001	0.44 (0.39–0.49)	0.68 (0.56–0.80)
High SES (vs other categories)	< 0.001	< 0.001	1.84 (1.68–2.03)	1.12 (1.09–1.16)
Age (6–12 years vs 12–15 years)	< 0.001	0.54	0.87 (0.81–0.94)	0.82 (0.43–1.56)
Father’s participation in OPA * Mother’s participation in OPA	0.03	0.06	1.16(1.05–1.40)	1.25 (0.99-1.46)

Significant associations in both school level groups were observed for gender and father’s and mother’s participation in an OPA. Age is at risk because it’s a highly significant *p* value. (*p* < 0.001) for non-participation in an OPA. Participation in sports tends to decrease with age even within primary school children.

As can be seen on Table 3, boys were more likely to participate in OPA than girls at both school levels. Regarding socio-economic status, children with low socioeconomic levels participated less frequently in OPA than children with a medium/high one and this association was highly significant (p < 0.001). Furthermore, regarding the impact of the area of residence, secondary schoolchildren living in an urban area participated more frequently in an organized physical activity than those living in a rural or semi-urban area in both school levels and these differences are highly significant (p < 0.001).

### Interaction effects

Significant interactions (*p* = 0.03) were observed between father’s and mother’s participation in OPA in primary school children. (unadjusted OR: 1.16; 95% CI: 1.05–1.40). Statistical interactions among all other potential covariates were tested, but none was significant.

### Μultivariate models

Table 4 shows the final results from the multivariate analysis of the factors associated with OPA at α = 5%. After adjustments for all potential covariates, being male and having a high SES seems to favor participation in an OPA in both school levels but to a different extent. Primary school children with low SES were 2.30 (95% CI:2.05–2.58) times more likely not to participate in OPA than other SES categories. Secondary schoolchildren were 1.49 (95% CI:1.31–1.70) times more likely not to participate in OPA than other SES categories. The father’s or mother’s participation in physical activity remained significant in a multivariate model for both school levels (*p* < 0.05). The interaction between the father’s and mother’s participation in OPA no longer affected primary schoolchildren’s participation in OPA in the multivariate model (*p* = 0.63). Also, the geographic area remained significant only for adolescents (*p* = 0.001). Being adolescent and residing in a rural area remained a significant factor for non-participation in OPA. Different categories of BMI by school level were associated with no participation in OPA. Being adolescent and underweight (*p* = 0.04) or a primary school pupil and obese (p = 0.04), increases the odds for non-participation in OPA.

**Table 4:** Multivariate analysis of covariates associated with OPA prevalence in the primary and secondary schools children, Greece

	*P* value		OR (95% CI)^a^	
Variables	Primary schoolchildren	Secondary schoolchildren	Primary schoolchildren	Secondary schoolchildren
Gender (males vs females)	< 0.001	0.01	1.25 (1.15–1.37)	1.17 (1.05–1.30)
Father’s participation in OPA (yes vs no)	< 0.001	0.03	1.52 (1.310–1.770)	1.16 (1.01–1.32)
Mother’s participation in OPA (yes vs no)	< 0.001	0.02	1.79 (1.31–1.77)	1.22 (1.03–1.43)
Obesity (vs normal weight)	0.036	0.37	0.75 (0.58–0.98)	0.91 (0.74–1.12)
Underweight (vs normal weight)	NS^a^	0.04	NS	0.76 (0.59–0.99)
Geographic area (Urban vs rural)	NS	< 0.01	NS	1.22 (1.09–1.36)
Low SES (vs other categories)	< 0.001	< 0.001	0.65 (0.58–0.73)	0.67 (0.59–0.77)
High SES (vs other categories)	< 0.001	< 0.01	1.38 (1.29–1.48)	1.36 (1.11–1.36)
Age (6–12 years vs 12–15 years)	0.03	NS	0.90 (0.83–0.98)	NS

The initial logistic models included, in addition to these, the categorical variable overweight (vs normal weight) for secondary schoolchildren and the interaction between father and mother’s participation in OPA in primary school children

^a^Non significant

Age remained a risk factor for non-participation in OPA within primary schoolchildren (*p* = 0.02). As a child grows older, from the first grades of primary school to the upper ones, they participated less frequently in OPA.

## Discussion

Developing of a better understanding regarding the magnitude of effect of potential factors associated with participation in OPA. BMI could affect participation through its effects on performance motivation and confidence. Longitudinal study has addressed the question whether there is an association between OPA and BMI and to which direction if there is one. They concluded that there is a bidirectional relationship, meaning that past participation in oPA predicts BMI and past BMI predicts participation in OPA [26]. It has been advised by recent guidelines as an intervention for overweight and obese children and adolescents [27].

This large cohort, nationwide study among school-age children in Greece showed that there was a high percentage of inactive children and adolescents, and this was affected by age, gender, BMI category, parent’ level of physical activityand socioeconomic factors.

BMI emerged as an important factor influencing participation in OPA. Weight status was significantly related to OPA, particularly for primary schoolchildren. Healthy-weight primary schoolchildren were more active than obese primary schoolchildren. As obesity is the result of a positive calorie balance it is contemplated that obese children are less physically active. Cadogan et al. [6] found an association between being overweight or obese and not achieving high levels of physical activity in a sample of Irish children. Similarly, Tambalis et al. [28], in a large epidemiological study of 8 to 9 year-old children, found a negative association between excess weight and performance in aerobic and motor fitness tests, but not in upper body strength. This could be related with the feelings of incompetence in physical activities, or unfavorable self-perceptions derived from social comparison with normal-weight peers [29]. Although some studies, as the Australian one [8] did not find any association between organized sports participation and BMI [7], the results of the present study are in agreement with previous findings showing an association of organized sports participation with BMI category. This study, further shows that this association remains significant even after controlling for age and family-related confounding variables. These results suggest that OPA participation is an important aspect when examining obesity, even at this young age. Regular participation in organized sports, already reduced the odds for being obese by almost 33% in primary schoolchildren. As this is a cross sectional study we cannot assert causality from the results of this study. It is not possible to understand whether this is a contributing factor to the development of overweight and obesity due to positive caloric balance or this is a consequence of the obesity as obese children are generally less competent in sports as a result of physical limitations or poor self-esteem. This is an important issue that needs to be dealt with and OPA should not only be addressed to strong athletes but offer the opportunity to all children to exercise in a noncompetitive manner.

An interesting and underreported finding is that underweight adolescents participate less in organized sports. In a sample of Polish adolescents (14–16 years old), found that underweight and overweight boys were characterized by significantly lower levels of physical activity compared to normal weight participants [30]. Vella et al. [8],, reported lower levels of sports participation among underweight adolescents in Australia and suggested that a possible explanation could be their low muscle mass, making them less successful when competing in sports, compared to normal-weight children. Nevertheless, the association between reduced sports participation, physical activity, and BMI category, is bidirectional and it is difficult to draw causality conclusions. An important association that emerged from this studyis the influence of the age group, as participation in OPA decreases dramatically from primary to high school: A large decrease in sports participation was noted as children went through adolescence. Lower levels of participation in OPA with increasing age were also reported for a multinational sample of British adolescents [31] and in Australian youth but to a lesser degree [32]. Possible causes for this decline in physical activity are changes in personal preferences, limited free time due to increased school obligations, increased need for socializing and going out with friends, body changes altering success rates in competitive sports.

With respect to gender, lower levels of sports participation among girls as compared to boys have been well established [31, 33, 34] and especially in OPA [7]. Results of our study are in the same direction. A possible explanation for the lower level of sports participation and physical activity in females could be related with the stereotype and gender roles set by society, which are internalized by early childhood through social influences by parents and grand parents and could determine perceived competence [35].

. Parents as a role model is a significant contributor to children’s involvement in OPA. Parents who are physically active, appeared to have a strong role in supporting children to be more active in both school levels. A number of studies support these findings. Cross-sectional researches in preschool and school children and their parents showed that parents whoare physically active, are more likely to support their children’s engagement in physical activity [36, 37]. Possible mechanisms for the relationship between parents’ and child’s activity levels include the parents’ serving as role models, enhancement and support by active parents of their child’s participation in physical activity, and genetically transmitted factors that predispose the child to be more physically active [38].Furthermore, the Greek family fits the ‘Mediterranean model’and consequently, it shares similar socio-demographic characteristics with other Mediterranean countries, meaning that traditional values and roles (marriage, family ties) are prevalent [39]. Thus, parents have a sound effect on their children’s every day habits.

The importance of socioeconomic status in influencing the participation in organized physical activity has been recognized in the present study, of Greek students.

These results may be partly explained by the social processes which occurred in Greece as a result of:

1. The financial crisis, when a significant number of pupils

transferred from private to public schools. The number of students in private education decreased to 70.7 thousand in 2015 from 94.2 thousand in 2000 and 93.4 thousand in 2009. Private schools lost more than 20,000 students (− 9.5%). It was observed a downward trend in higher income scales and a corresponding quadrupling of the lower income category [40].

2. The increasing educational level of Greeks and Greek women in particular [40, 41]

Our findings that socioeconomic status is a significant predictor of participation in OPA in both school levels, are consistent with reports for children and adolescents of other countries [17, 42–45]. Parents from higher socioeconomic classes will probably encourage their children to participate in OPA to a greater extent than parents from lower socioeconomic classes. Such findings may be explained by the fact that some OPA are not easily accessible by lower socioeconomic classes because of the lack of transport or money.

Additionally, the area of residence impacts on the level of participation in OPA. The results of this study showed higher level of participation in OPA of the residents of urban settings. This finding contrasts with previous reports of other countries stating that children residing in cities engage in OPA much less as compared to their peers residing in rural areas [8, 15, 46, 47]. At this point, it should be noted that the lack of infrastructure in rural areas, and the small number of inhabitants, preclude the access of adolescents to gyms and sports facilities.

Limitations of the present study arethe self-reporting of information regarding sports participation which is a source of bias and the cross-sectional design which does not allow for causality conclusions. Thus, temporal relationship between OPA participation and covariates is unknown. Longitudinal data are necessary to further unravel the complex interplay between the endogenous variable and the above mentioned covariates. Strong points of the study are the representative sample, the nationwide participation, the large number of the cohort that increase the external validity of the study.

## Conclusions

Participation in OPA decreases with age, the greatest difference being observed after transition from primary to secondary school. Significant factors associated with the lack of physical activity were: the low socioeconomic status, the female gender, the BMI category (obese and underweight, for primary and secondary schools respectively), the parents’ non participation in OPA, being adolescent and residing in a rural area. This study provides additional evidence that the family constitutes an important socializing agent and role model regarding OPA. Intervention programs’ designers are encouraged to develop methods that will aim to parental motivation in order to improve OPA participation of all family members. Parents provide a target group for interventions, especially at early school ages, in order to understand the importance and be motivated to promote the importance of PA in their children either by acting as model behaviour or by supporting their child’s active participation to sport activities.

Covariates that are not considered modifiable, such as age and gender, can be used to guide targeted interventions and policies.

Finally, a better understanding of trends in children’s physical activity patterns within different weight status, SES areas and regional environments are the challenges faced by municipalities. The delivery of increased hours of physical education and sports incorporated into school daily program can possibly eliminate the impact of SES inequalities. Longitudinal studies will further elucidate these issues.

## Data Availability

The datasets generated during and/or analysed during the current study are available from the corresponding author on reasonable request.

## Abbreviations

BMI: Body Mass Index
OR: Odds Ratio
OPA: Organized Physical Activity
